# Regional Medical Collaboration May Lead to Early Detection of Interstitial Lung Disease

**DOI:** 10.3390/jcm14175923

**Published:** 2025-08-22

**Authors:** Yoshiaki Zaizen, Masaki Tominaga, Goushi Matama, Yutaka Ichikawa, Rumi Gohara, Junichiro Hiyama, Souichiro Ide, Tomoko Kamimura, Masaharu Kinoshita, Yasuhiko Kitasato, Takeharu Koga, Yousuke Miyagawa, Hideo Ogino, Rumi Sato, Yoshiko Sueyasu, Kazuhiko Yamada, Tomoaki Hoshino

**Affiliations:** 1 Division of Respirology, Neurology and Rheumatology, Department of Medicine, Kurume University School of Medicine, 67 Asahi-machi, Kurume 830-0011, Fukuoka, Japan; tominaga_masaki@med.kurume-u.ac.jp (M.T.);; 2 Department of Pathology Informatics, Nagasaki University Graduate School of Biomedical Sciences, 1-7-1 Sakamoto, Nagasaki 852-8501, Japan; 3 Department of Community Medicine, Kurume University School of Medicine, 67 Asahi-machi, Kurume 830-0011, Fukuoka, Japan; 4 Gohara Clinic, 608-2 Naganobu, Hirokawa 834-0105, Fukuoka, Japan; 5 Hiyama Clinic, 1020-2 Oosaki, Ogouri 838-0127, Fukuoka, Japan; 6 Department of Respiratory Medicine, Imamura Hospital, 1523-6 Todoroki-machi, Tosu 841-0061, Saga, Japan; 7 Department of Respiratory Medicine, Yame General Hospital, 540-2 Takatsuka, Yame 834-0034, Fukuoka, Japan; 8 Department of Respiratory Medicine, Nagata Hospital, 523-1 Shimomiyanaga-machi, Yanagawa 832-0059, Fukuoka, Japan; 9 Department of Respiratory Medicine, Japan Community Healthcare Organization, Kurume General Hospital, 21 Kushiwara-machi, Kurume 830-0013, Fukuoka, Japan; 10 Koga Clinic for Internal and Respiratory Medicine, 1880-8 Miyano, Asakura 838-1302, Fukuoka, Japan; 11 Department of Respiratory Medicine, Koga Hospital 21, 3-3-8 Miyanojin, Kurume 839-0801, Fukuoka, Japan; 12 Hita Ishii-machi Clinic, 580-1 Ishii, Hita 877-0061, Ooita, Japan; 13 Department of Respiratory Medicine, Asakura Medical Association Hospital, 422-1 Raiha, Asakura 838-0069, Fukuoka, Japan; 14 Department of Respiratory Medicine, Saiseikai Futsukaichi Hospital, 3-13-1 Yumachi, Chikushino 818-8516, Fukuoka, Japan; 15 Department of Respiratory Medicine, Shin Koga Hospital, 120 Tenjin-machi, Kurume 830-8577, Fukuoka, Japan

**Keywords:** interstitial pneumonia, interstitial lung disease, idiopathic pulmonary fibrosis, medical cooperation system, regional collaboration

## Abstract

**Background**: Establishing a highly accurate regional medical collaboration (RMC) system for interstitial lung disease (ILD) may facilitate early disease detection, improve patient satisfaction, and enhance advanced-stage care. **Methods**: We investigated whether the lung conditions in patients cared for by our RMC system for ILD were detected earlier than those with usual care. Additionally, we investigated patients’ preferences regarding its use. **Result**: The time from respiratory symptoms onset to hospital referral did not differ significantly between patients cared for by the system and those with usual care. However, the number of patients referred to our hospital for suspected ILD before the onset of symptoms was significantly higher from the participating institutions than from other institutions (44.1% vs. 24.6%, *p* = 0.025). Additionally, 66.0% of patients preferred the medical care with the system. **Conclusions**: Establishing an RMC system for ILD may lead to earlier disease detection and contribute to improvement in medical care delivery to patients.

## 1. Introduction

Interstitial lung disease (ILD) is a large and heterogeneous group of diseases characterized by inflammation and fibrosis, primarily in the lung interstitium [1]. The most common form of ILD is idiopathic pulmonary fibrosis (IPF); however, this group of diseases includes IPF and a group of diseases called progressive pulmonary fibrosis (PPF), in which fibrotic lesions of the lungs present with chronic progressive respiratory failure, and in numerous cases, a poor prognosis with a declining quality of life (QOL) [2,3]. Currently, antifibrotic therapy is the accepted treatment for IPF and PPF. The antifibrotic agents pirfenidone and nintedanib have been shown to reduce the decline in forced vital capacity (FVC) in patients with IPF [4,5]. In addition, antifibrotic therapy has been associated with a reduced risk ratio for all-cause mortality in IPF, which suggests that it may contribute to prolonged survival [6,7]. In ILD other than IPF, nintedanib has been shown to reduce the decline in FVC in a group of diseases with progressive fibrosing ILD, which is similar to PPF [8]. However, the efficacy of pirfenidone and nintedanib is to reduce the progression of these diseases, not to improve them. Antifibrotic therapy has also been shown to reduce the decline in FVC in relatively early stage IPF patients with preserved FVC [9]. Therefore, early initiation of pharmacologic therapy in IPF and PPF to control disease progression is important to preserve the prognosis [10]. Moreover, pulmonary rehabilitation has been shown to increase 6-min walk distance, reduce dyspnea, and improve quality of life in patients with ILD [11]. However, these benefits have been shown to be less effective in more severe patients, such as those with exercise hyperoxemia. Pulmonary rehabilitation in early stage ILD patients is also important to preserve the QOL [12].

Diagnosing patients with fibrotic ILDs is often delayed due to the subtle and nonspecific nature of symptoms in the disease’s early stages [13,14]. Hoyer et al. show that there is not only a significant patient delay before IPF patients are admitted to a clinic, but also a significant diagnostic delay caused by general practitioners (GPs) and community hospitals, with an overall diagnostic delay of 2.1 years before IPF patients receive an appropriate diagnosis [13]. Patient unawareness or lack of knowledge is one of the major reasons for delays in diagnosing patients with fibrotic ILDs [14]. In addition, misdiagnosis by primary care physicians or physicians without expertise in ILDs is also among the major reasons for delays [14,15]. In patients with these fibrotic ILDs, particularly because of the progressive and often irreversible nature of the conditions, early diagnosis of the disease is crucial, along with support to those experiencing progressive chronic respiratory failure that affects their QOL [16]. This requires medical collaboration involving not only specialized ILD centers but also regional hospitals, local pulmonologists, and GPs as a part of a community-based healthcare network.

We established a regional medical collaboration (RMC) system to support patients with ILD. In this study, we investigated whether the RMC system resulted in the early detection of ILD in patients referred to our hospital. We also made a questionnaire that was given to the patients to determine the need for such an RMC system among patients with ILD.

## 2. Materials and Methods

### 2.1. Establishment of Regional Medical Collaboration System

We established an RMC system to support patients with ILD in the Chikugo area of Fukuoka Prefecture, an area of approximately 1500 square kilometers and a population of approximately 830,000 people in Japan, led by doctors who are board-certified members or senior fellows of the Japanese Respiratory Society. In this system, Kurume University Hospital, a specialized ILD center, collaborates with 15 local general hospitals and 22 local GPs with expertise in respirology to treat patients with ILD. Fourteen out of 15 local general hospitals and 21 out of 22 local GPs in the RMC system have respiratory specialists on staff who are board-certified members or senior fellows of the Japanese Respiratory Society. One additional local general hospital has a collagen disease specialist who is primarily responsible for treating ILD associated with collagen disease. All referrals from these hospitals and general practitioners to Kurume University Hospital are made at the discretion of the specialists working at these facilities, and all referred patients are cared for at Kurume University Hospital. At Kurume University Hospital, even when a patient was referred to Kurume University Hospital for the first time, if the referral was made through the RMC system, the patient was directed to the outpatient clinic specializing in ILD from the beginning. Responsibilities are shared, including comprehensive examinations, disease assessments, routine drug therapy including immunosuppressants or antifibrotic agents, management of chronic respiratory failure, and end-of-life care. To share this information, we also created a notebook that patients were asked to bring with them when they visited the RMC system hospital and that each physician used as a medical record. We share information with Kurume University Hospital and local hospitals or GPs, and patients basically visit Kurume University Hospital once every three months for chest radiography and pulmonary function tests to assess their health status. In addition, local hospitals and GPs check in with patients about once or twice a month for health monitoring, prescription of medications, and necessary vaccinations. If the patient’s condition worsens, this notebook entry replaces the medical information form, reducing the referral burden on physicians at local hospitals or GPs. Additionally, semiannual liaison meetings and study sessions were held with collaborating physicians in the RMC system to maintain the quality of ILD care.

We are the first in our country to establish and operate such an RMC system dedicated to ILDs since October 2022. Until we established the RMC system, the workflow for referring ILD patients to Kurume University Hospital was the same as for other diseases: patients were sent to the hospital’s regional medical coordination office for a referral letter, and then an appointment was made to consult at Kurume University Hospital. In addition, when patients first visited Kurume University Hospital, they were first examined by a respiratory physician who did not specialize in ILD and then referred to an outpatient clinic that specialized in ILD. In addition, if a patient who was being treated at Kurume University Hospital was referred from a local clinic or general hospital for any reason, he or she had to contact the regional medical coordination office at Kurume University Hospital and make an appointment in the same way.

In summary, the RMC system simplifies the conventional referral procedure and allows referrals from cooperating physicians who have received information exchange and education meetings in cooperation with the RMC system to be referred directly to the ILD Specialist Outpatient Clinic at Kurume University Hospital. In addition, patients will be cared for collaboratively by the physicians at Kurume University Hospital and the cooperating physicians. The procedure for emergency visits to Kurume University Hospital in the event of a patient’s deterioration has also been simplified.

### 2.2. Study Participants

In this study, the period from April 2022 to September 2022 was set as the period before the start of the RMC system. The period from October 2022 to September 2023 was set as the period after the start of the RMC system. Patients referred to Kurume University Hospital with suspected ILD during these periods were included. The background of these patients was investigated using their medical records. The study examined the presence or absence of symptoms at the time of referral to Kurume University Hospital, as well as the time from the first awareness of symptoms to the referral.

The diagnosis of ILD in the current study population was based on the guidelines for IPF, hypersensitivity pneumonitis, and connective tissue disease-associated interstitial lung disease [1,2,17,18]. Transbronchial lung cryobiopsy was proposed for patients who did not have a typical or probable UIP pattern on HRCT in the guideline [2] and did not clearly meet the diagnosis of collagen disease, and transbronchial cryobiopsy was performed in those patients who agreed. Transbronchial lung cryobiopsy was performed in 48 patients (37.5%). The diagnosis of ILD was made by multidisciplinary discussion with radiologic and pathologic information.

### 2.3. Patient Questionnaire Surveys

We administered a five-point questionnaire to the study participants to obtain information on the acceptance of the care delivered by the system. We asked the following five questions in this questionnaire:

Q1. Do you think the referral to Kurume University Hospital (from a visit to a local clinic to a visit to the University Hospital) went smoothly?
Q2. When you first visited Kurume University Hospital, did you think your examinations and consultations were performed smoothly?
Q3. Do you think your subsequent examinations and treatment (including follow-up) at Kurume University Hospital were carried out smoothly?
Q4. Do you think there is sufficient cooperation between local clinics/hospitals and Kurume University Hospital?
Q5. If you are going to receive treatment in the future, would you prefer to be treated in the ILD regional medical collaboration system? In this regional medical collaboration system, patients visit the university hospital once every few months for evaluation of their medical conditions and to determine the treatment plan, and then they visit their local family doctor’s office once a month for health observation and treatment based on the university hospital’s plan.

We asked them to respond to these five questions by choosing from the following five responses: Strongly agree (5), Agree (4), Neutral (3), Disagree (2), and Strongly disagree (1). We obtained verbal and written consent for the survey study from subjects who continued to attend the outpatient clinic at Kurume University Hospital and collected the questionnaires using collection boxes. The questionnaires were collected for about four months. The responses (scores) for each item were collected from the collected questionnaires.

### 2.4. Statistical Analyses

Statistical significance of the difference between the two groups was analyzed using Fisher’s exact test for the patient’s sex or the number of patients without respiratory symptoms, Wilcoxon rank-sum test for the patient’s age or the duration from symptom onset to referral, and Cochran–Armitage test for trends in the patient’s diagnosis or the five-point questionnaire results. Statistical significance was defined as *p* < 0.05, with all statistical analyses performed using JMP 16.0 (SAS Institute, Cary, NC, USA).

## 3. Results

### 3.1. Patient Characteristics

Table 1 shows the patients’ background. A total of 128 patients were included in the study, with a median age of 72 years (62–76), and 69 (53.9%) were male. Thirty-three patients (25.8%) were included earlier in the study, and 95 patients (74.2%) were included after the start of the RMC system. Of the 95 patients, 59 (62.1%) were referred by respiratory physicians from hospitals or GPs in cooperation with the RMC system. Forty-three patients (33.6%) had no respiratory symptoms at the time of referral to our university hospital (e.g., abnormal findings during medical checkups). All patients were referred to our university hospital with suspected ILDs, including those diagnosed by multidisciplinary discussion: 29 (22.7%) with IPF, 20 (15.6%) with hypersensitivity pneumonitis, and 35 (27.3%) with connective tissue disease-related interstitial pneumonia. Additionally, 11 (8.6%) patients were referred to our hospital due to suspicion of ILDs but were diagnosed with conditions other than ILDs, such as lung cancer, pulmonary tuberculosis, or interstitial lung abnormalities.

### 3.2. Early Detection of Patients with ILD and RMC

Table 2 compares the time of patient referral before and after the start of the RMC system and whether the patient was referred by a cooperating hospital/GP in the RMC system. No difference was observed in the duration from when patients showed respiratory symptoms to when they were referred to our hospital, whether it was before or after the start of the RMC system, or whether they were referred by hospitals or GPs cooperating with the RMC system. However, 26 patients (44.1%) were referred by cooperating hospitals/GPs before presenting respiratory symptoms, which was significantly higher than the 17 patients (24.6%) referred by other hospitals/GPs (*p* = 0.025).

### 3.3. Patient Questionnaire on ILD RMC

We received questionnaires from 48 (37.5%) of all 128 patients, including 25 patients who were referred from the cooperating hospital/GP in the RMC system. All of the responses to the survey are listed in Table 3.

**Figure 1:**
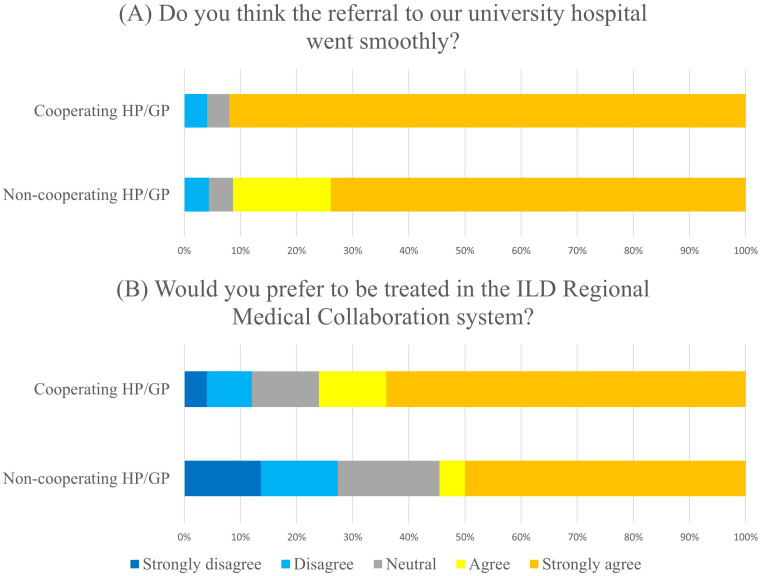
Patient Questionnaire on Interstitial Lung Disease Regional Medical Collaboration. (**A**) Do you think the referral to our university hospital went smoothly? Strongly agree and agree in the patients from cooperating HP/GP was 91.3%, and from non-cooperating HP/GP was 92.0% (*p* = 0.368). (**B**) Would you prefer to be treated in the ILD Regional Medical Collaboration system? Strongly agree and agree in the patients from cooperating HP/GP was 76.0%, and from non-cooperating HP/GP was 54.5% (*p* = 0.136).

Among them, 44 of 48 (91.7%) strongly agreed or agreed that referral to our university hospital was smooth and that no difference was observed as to whether they were referred from a cooperating hospital/GP with the RMC system (*p* = 0.368, Figure 1). On the other hand, 31 patients (66.0%, one non-response) strongly preferred or preferred to be treated in the RMC system at both the university hospital and community hospitals/GPs, while nine patients (19.1%) opposed or strongly opposed it. Patients referred by hospitals/GPs cooperating with the RMC system were more willing to be treated using the RMC system (76.0% vs. 54.5%), but the difference was not statistically significant (*p* = 0.136).

## 4. Discussion

In this study, we found that our RMC system for ILDs led to an increase in the proportion of patients with ILD who were referred before symptom onset, thereby contributing to the early detection of ILDs. Our RMC system is characterized by a regular half-yearly information exchange meeting, which includes new information about ILDs. Cosgrove et al. reported that 55% of patients with ILD had at least one or more different diagnoses before being diagnosed with ILD [15]. If we succeed in establishing a highly accurate RMC system that effectively disseminates ILD-related information to clinicians in local hospitals and GPs, we can anticipate a reduction in diagnostic delays for ILDs. In addition, our RMC system features collaboration between ILD specialty centers and local hospitals and GPs in the daily care of each patient. Regional healthcare systems are certainly subject to significant restrictions depending on the country or region in which they operate. Therefore, it is not possible to definitively conclude whether the ILD-RMC system is useful for early detection nationwide based on this study alone. However, it may be possible to conclude based on this study that the system of regular collaboration between ILD specialty centers and local hospitals and GPs has the potential to lead to referring ILD patients to ILD specialty centers without feeling any obstacles, as well as to the early detection of ILD patients.

Additionally, RMC for ILD was highly desired by 66% of the patients in this study, suggesting a high demand among patients with ILD. In our system, patients visit Kurume University Hospital, a specialized ILD center, once every three to six months, and in the meantime, routine examinations and routine prescriptions and vaccinations are performed at local hospitals and clinics. We believe that promoting RMC for ILD can improve medical care delivery to affected individuals. In addition, we also believe this will reduce the burden of hospital visits on patients and help reduce the environmental burden associated with such visits.

The major limitation of this study is that it analyzed only a single system located in one region of Japan. Healthcare systems differ from country to country and region to region, and our system may not necessarily fit the circumstances of each region. However, there is no doubt that patients will benefit greatly from a healthcare system that is tailored to each country. This is the point we want to emphasize in this study, and the fact that it also has some impact on ILD, which is an incurable disease. In addition, this study only evaluated patients for 6 months prior to the establishment of the RMC system. This leaves us with a small number of patients before the RMC system was established. This was a problem with our medical record system. Another limitation of this study is that only 37.5% of the questionnaires were collected, which is not an evaluation of everyone who received medical care through the RMC system.

## 5. Conclusions

The establishment of the RMC system for ILD has the potential to lead to early detection of ILD patients. In addition, such an RMC system may be in high demand by patients. RMC in ILD should be further encouraged in the future.

## Figures and Tables

**Table 1:** Patient characteristics.

	Total	Before ILD-RMC System	After ILD-RMC System	*p*-Value
Number	128	33 (25.8%)	95 (74.2%)	
Age ^†^	72 (62–76)	69 (63–78)	72 (62–76)	0.657
Sex: Male	69 (53.9%)	17 (51.5%)	52 (54.7%)	0.840
Cooperating hospital/GP	59 (62.1%)	N/A	59 (62.1%)	
Non-cooperating hospital/GP	36 (37.9%)	N/A	36 (37.9%)	
Symptoms				
Present	85 (66.4%)	25 (75.8%)	60 (63.2%)	0.207
Absent	43 (33.6%)	8 (24.2%)	35 (36.8%)	
Days from onset of symptom to referral *	30 (11–134)	34 (13–113)	28 (11–168)	0.777
Diagnosis				0.803
IPF	29 (22.7%)	5 (15.2%)	24 (25.3%)	
Unclassifiable IIPs	20 (15.6%)	4 (12.1%)	16 (16.8%)	
Other IIPs	9 (7.0%)	4 (12.1%)	5 (5.3%)	
Hypersensitivity pneumonitis	20 (15.6%)	5 (15.2%)	15 (15.8%)	
RA-ILD	5 (3.9%)	3 (9.1%)	2 (2.1%)	
SSc-ILD	9 (7.0%)	3 (9.1%)	6 (6.3%)	
PM/DM-ILD	15 (11.7%)	5 (15.2%)	10 (10.5%)	
Other CTD-ILD	6 (4.7%)	2 (6.1%)	4 (4.2%)	
Drug-induced ILD	3 (2.3%)	1 (3.1%)	2 (2.1%)	
Eosinophilic pneumonia	1 (0.8%)	0 (0.0%)	1 (1.1%)	
Not ILD ^‡^	11 (8.6%)	1 (3.1%)	10 (10.5%)	

CTD, connective tissue disease; GP, general practitioner; IIPs, idiopathic interstitial pneumonias; ILD, interstitial lung disease; IPF, idiopathic pulmonary fibrosis; PM/DM, polymyositis/dermatomyositis; RA, rheumatoid arthritis; RMC, regional medical collaboration; SSc, systemic sclerosis. * Excludes patients who have already been treated at the referral site. ^†^ Median values with 25–75% of the interquartile range. ^‡^ “Not ILD” includes interstitial lung abnormality.

**Table 2:** The usefulness of the RMC system for early detection of ILD.

	Before ILD-RMC System	After ILD-RMC System		*p*-Value ^†^	*p*-Value ^‡^
		Non-Cooperating Hospital/GP	Cooperating Hospital/GP		
Number	33	36	59		
Respiratory symptom absent	8 (24.2%)	9 (25.0%)	26 (44.1%)	0.207	0.025
Days from onset of symptom to referral *	34 (13–113)	33 (10–330)	24 (11–96)	0.777	0.320

GP, general practitioner; ILD, interstitial lung disease; RMC, regional medical collaboration. ^†^ Before ILD-GMC system vs. after ILD-GMC system; ^‡^ cooperating hospital/GP vs. others. * Excludes patients who have already been treated at the referral site.

**Table 3:** Results of the patient questionnaire survey.

		5	4	3	2	1
Q1.	Total	40	4	2	2	0
	Cooperating HP/GP	23	0	1	1	0
	Non-cooperating HP/GP	17	4	1	1	0
Q2.	Total	40	5	2	1	0
	Cooperating HP/GP	21	2	1	1	0
	Non-cooperating HP/GP	19	3	1	0	0
Q3.	Total	44	4	0	0	0
	Cooperating HP/GP	23	2	0	0	0
	Non-cooperating HP/GP	21	2	0	0	0
Q4.	Total	32	10	5	1	0
	Cooperating HP/GP	18	5	2	0	0
	Non-cooperating HP/GP	14	5	3	1	0
Q5 *.	Total	27	4	7	5	4
	Cooperating HP/GP	16	3	3	2	1
	Non-cooperating HP/GP	11	1	4	3	3

GP, general practitioner; HP, hospital. * There was one person who did not answer this question.

## Data Availability

The datasets used and/or analyzed during the current study are available from the corresponding author on reasonable request.

## Abbreviations

The following abbreviations are used in this manuscript:

RMC	Regional medical collaboration
ILD	Interstitial lung disease
IPF	Idiopathic pulmonary fibrosis
PPF	Progressive pulmonary fibrosis
QOL	Quality of life
GPs	General practitioners
